# Design and development of an eHealth intervention to support self-management in people with musculoskeletal disorders - ‘eHealth: It’s TIME’: a study protocol

**DOI:** 10.12688/hrbopenres.13611.2

**Published:** 2023-08-10

**Authors:** Marie Kelly, Brona Fullen, Denis Martin, Colin Bradley, Billy O'Mahony, Joseph G. McVeigh

**Affiliations:** 1 Discipline of Physiotherapy, University College Cork, Cork, Ireland; 2 Department of Physiotherapy, Mercy University Hospital, Cork, Ireland; 3 School of Public Health, Physiotherapy and Sports Science, University College Dublin, Dublin, Ireland; 4 School of Health and Social Care, Teesside University, Middlesbrough, UK; 5 Department of General Practice, University College Cork, Cork, Ireland; 6 School of Computer Science and Information Technology, University College Cork, Cork, Ireland

**Keywords:** eHealth, self-management, intervention development, musculoskeletal pain, user-centred design approach

## Abstract

**Background**: Musculoskeletal disorders (MSDs) are a leading cause of global morbidity, with the burden expected to increase in the near future. Self-management, with the support of healthcare professionals, is recommended for many MSDs. However, frequent clinical contact is not feasible. Previous research has highlighted the need for a co-designed eHealth-mediated self-management follow-up support intervention which integrates remote monitoring and behavioural change. Thus, the current study aims to develop and design a user-centred, eHealth-mediated self-management support prototype for people with MSDs.

**Methods**: A three-step, iterative system development cycle will be utilised to develop and design the “eHealth: It’s TIME prototype”. The three-step process will include creating website features and content using two sequential focus groups with people with MSDs (n = 6 – 8); heuristic testing using the 10 heuristic principles of Nielsen (n = 5); and usability testing through in-person 60-minute interviews with people with MSDs (n = 3 – 5) and musculoskeletal physiotherapists (n = 3 – 5).

**Conclusion**: The eHealth: It’s TIME prototype will be a systematically developed, follow-up self-management support intervention guided by behavioural change theory and the preferences of end users.

## Introduction

Globally, musculoskeletal disorders (MSDs) are the leading contributor to the need for rehabilitation ^1^, with this intervention essential in maximising a person’s function and quality of life ^2^. Despite these individual and societal benefits, rehabilitation services remain under-resourced, and this, together with the high demand for such services, has created an enormous musculoskeletal (MSK) care gap ^1^. While this divide continues to widen, solutions to this challenge are urgently needed ^3^; solutions which deliver person-centred care and empower people with MSDs to self-manage their condition are critical ^3^. 

Self-management is a complex intervention that involves patient education and behaviour modification and is developed to give people the skills to address the physical, emotional, and social challenges associated with their condition ^4– 6^. Self-management requires a collaborative approach, in which the healthcare professional delivers ongoing support ^7^. Given limited healthcare resource availability, eHealth interventions are increasingly being viewed as the answer to improving MSK care access and supporting self-management ^8– 10^. However, while their use has been growing, with the coronavirus disease 2019 (COVID-19) pandemic being seen as a significant catalyst ^11^, sustaining this has remained elusive ^12^. A lack of user ( *i.e.* people with MSDs) involvement is considered a major contributing factor to this ^13^, resulting in usability issues and high dropout rates, and ultimately eHealth interventions that are “high tech-with-a-low impact” ^14, 15^.

 A recent scoping review reported only 22% (14/63) of the 63 eHealth-mediated self-management support interventions were co-designed ^16^, despite numerous calls to utilise a more user-centred design approach ^17– 19^. User-centred design is an approach that involves users in the development and decision-making process ^20^, resulting in interventions which demonstrate greater effectiveness, satisfaction and usability than traditionally developed (i.e., linear, often top-down) interventions ^21^.

This study is part of a larger project, ‘eHealth: It’s TIME’, which aims to develop an eHealth-mediated self-management support intervention for people with MSDs. For development processes and eHealth interventions to be successful, a holistic focus, encompassing the habits and rituals of the end users, intervention setting and context, is required ^22, 23^. This involves identifying barriers and facilitators and exploring implementation challenges at the intervention planning stage ^24, 25^. Hence, this study is informed by our earlier research utilising a systematic scoping approach to chart the current literature base ^16^ and establish stakeholder perceptions towards eHealth-mediated self-management support for people with MSDs ^26^. People with MSDs and MSK physiotherapists reported concerns about assessment and diagnosis and establishing a therapeutic relationship and felt that eHealth interventions may be best reserved for follow-up care ^26^. Considering these findings, this study in the ‘eHealth: IT’s TIME’ project aims to co-develop and design a prototype of an intervention that facilitates follow-up self-management support via integrated remote monitoring and behavioural change. This work will likely contribute to the development and design of a more easy-to-use, worthwhile intervention that can potentially improve treatment outcomes for people living with MSDs ^18, 22, 27^. The ‘eHealth: It’s TIME’ project is guided by the Medical Research Council (MRC) framework ^28^ and the CeHReS (Center for eHealth Research) Roadmap ^22, 29^.

## Methods

### Study aims

The present study aims to systematically develop and design a user-centred web-based prototype for adults with MSDs to enhance follow-up self-management support via integrated remote monitoring and behavioural change.

### Study design

**Figure 1.**
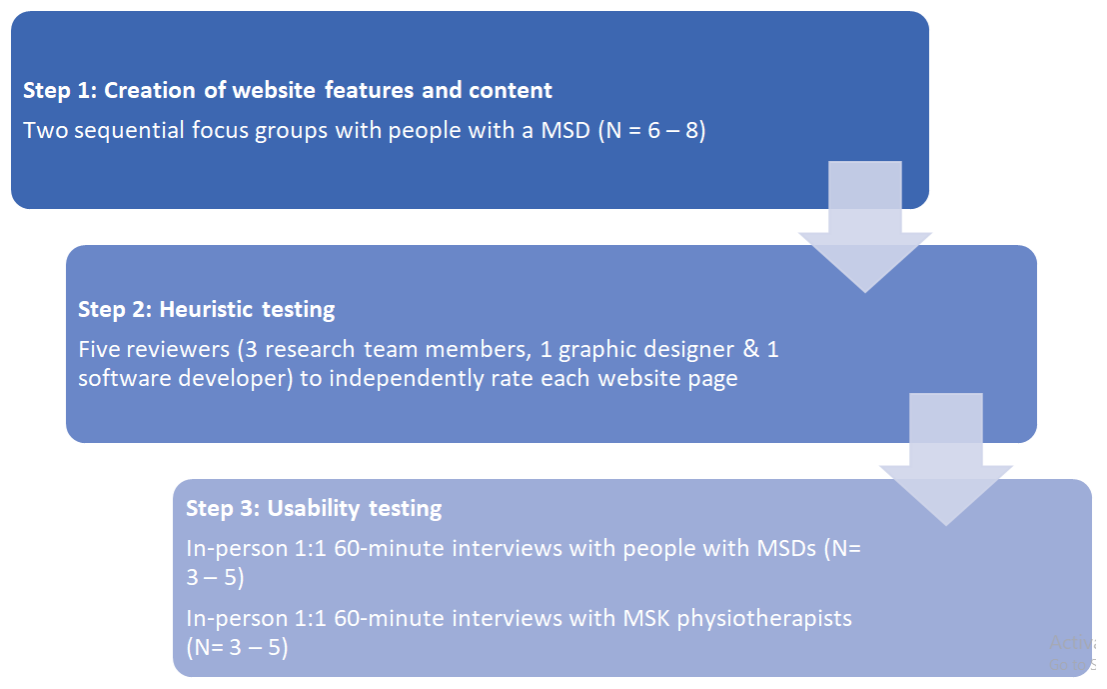
Study overview.

**Figure 2.**
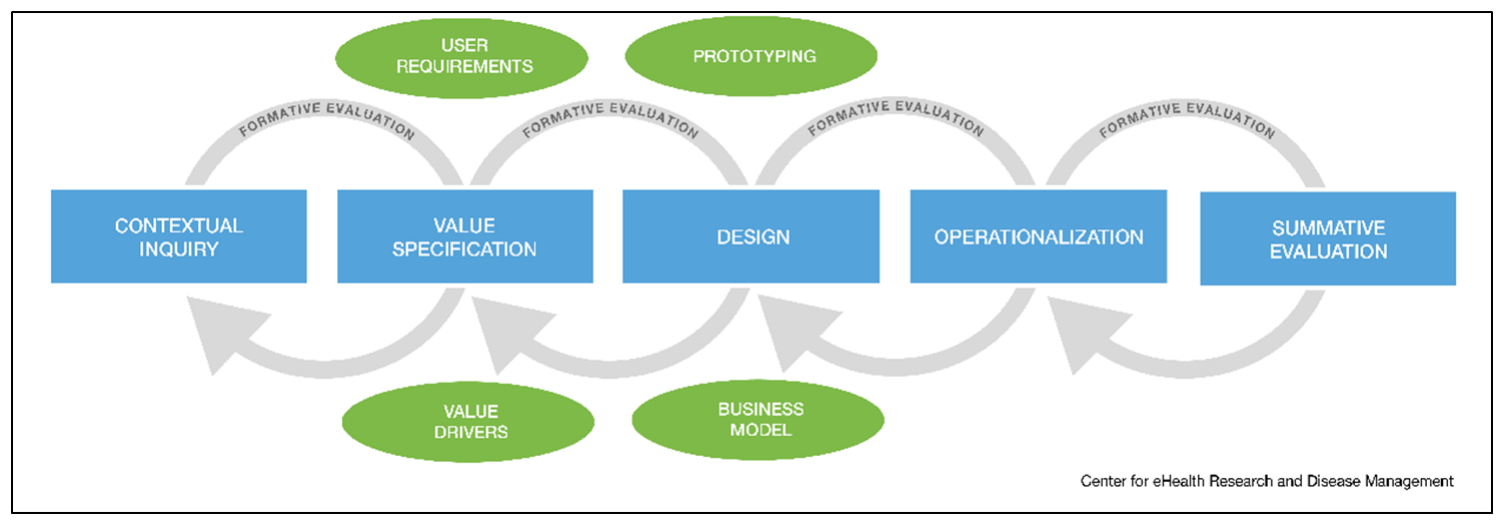
The CeHRes Roadmap (the Centre for eHealth Research Roadmap) ^22^.

The eHealth: It's TIME prototype will be co-developed and designed using a three-step system development cycle ^30, 31^ which includes the creation of website features and content (Step 1); heuristic testing (Step 2); and usability testing (Step 3) ( Figure 1). The CeHRes Roadmap ^22^ will inform the development and design process of the eHealth-mediated follow-up self-management support intervention. This evidence-based roadmap ^22^ ( Figure 2) provides practical guidance on how to execute the participatory eHealth development process, fulfilling the MRC framework criteria ^28^, with the latter framework providing limited information on how to design complex interventions ^32^.

The CeHRes Roadmap has been utilised for eHealth development in numerous settings ^33– 36^ and complex contexts ^22^, such as MSK healthcare. As the ‘eHealth: It’s TIME’ project relates to the development and design of eHealth technology (and not the implementation or evaluation), the focus lies on the first three phases of the Roadmap: contextual inquiry ^16^, value specification ^26^ and design (current study). 

The eHealth: It’s TIME development and design is being led by MK, a part-time PhD candidate and senior musculoskeletal physiotherapist. The focus groups and interviews will be conducted by MK who has undertaken qualitative research training. Other members of the research team include three academic physiotherapists (JMcV, BF, and DM), one academic General Practitioner (CB) and one software developer (BO’M); all of whom have qualitative research experience.

Ethical approval has been granted by the Clinical Research Ethics Committee (CREC) of the Cork Teaching Hospitals (REF: ECM 4 (f) 20/09/2022). The study will be performed in accordance with the Declaration of Helsinki.

### Step 1: Creation of website features and content

***Study participants***

Using convenience sampling, individuals with a MSD will be eligible to participate in this study if they are English speaking (judged by researcher MK during pre-interview communication); 18 years or older; and able to provide electronic or written informed consent. Participants with pain of specific pathological origin ( *e.g.* infection, malignancy, inflammatory disease or fracture); those that are pregnant; or have had surgery within the past six months; will be excluded. Initial eligibility screening for individuals with a MSD will be undertaken through the online platform Qualtrics. For those that do not have access to a computer or the internet, the screening will be conducted via post.

***Recruitment***

Individuals with MSDs will be recruited through various methods, with interested participants asked to contact the researcher MK:

- Primary and secondary care: The researcher MK will email physiotherapy managers and General Practices in Cork City outlining the details of the study and request that information about the study be circulated to all their staff to distribute to their patients with a MSD. A poster for display in waiting rooms will be included. At one hospital site (Mercy University Hospital), potential participants will be identified by MSK physiotherapy staff. Eligibility screening will be completed by the researcher (MK). Potentially eligible participants will be informed about the study by the lead researcher (MK) and will receive a participant information leaflet. Following a reflection period of 24h, the researcher will telephone the individual with the MSD to confirm their interest in participation.
- Professional Organisation: The call for participants along with the poster will be sent to physiotherapists via the Irish Society of Chartered Physiotherapists (ISCP) mailing list.
- Patient support groups: An email request will also be sent out to people with MSDs via gatekeepers in Arthritis Ireland and Chronic Pain Ireland.
- University staff: An email request will also be sent out to all academic, research and administrative staff in UCC, targeting those with a MSD via a gatekeeper.
- Social media: The study will also be advertised on social media (Twitter). Interested participants will be asked to contact the researcher MK.

Those with access to email that express interest in the study will be sent a participant information leaflet, and a Qualtrics link to the Consent form. For those that do not have access to a computer or the internet, the participant information leaflet and consent form will be sent via post with a prepaid return envelope. 

***Procedures***

**Questionnaires**

Before the focus group, participants with a MSD will complete two questionnaires via Qualtrics: (1) demographic details and (2) the eHealth Literacy Scale (eHEALS). The eHEALS is an eight-item self-report measure assessing individuals’ perceived ability to locate, evaluate, and apply eHealth information ^37^. The overall scores range from 8 to 40, with higher scores indicating higher self-perceived levels of eHealth literacy ^37^.

**Focus Groups**

Two sequential focus groups, involving between six and eight participants, will be facilitated by MK (lead researcher) to elicit users’ views and preferences regarding the proposed features. Focus groups are considered an appropriate method for informing intervention development ^38^. The focus groups will take place in person but can be held remotely if necessary ( *e.g.*, for COVID health and safety reasons) via Microsoft Teams. Each focus group is expected to last 60 minutes. In situations where non-attendance or scheduling difficulties occur, one-to-one interviews will be conducted utilising the same topic guide. At the outset of each focus group interview, informed consent will be re-confirmed orally, and audio-recorded digitally.

Following the scoping review ^16^ and qualitative study ^26^, a list of the individual with a MSD’s needs and requirements was formulated. Proposed eHealth intervention prototype elements to meet these needs ( **Appendix A, *Extended data***) will be built prior to the first focus group. While building these elements, the Web Content Accessibility Guidelines ^39^ will be followed, making content accessible to a wider range of people with disabilities ^40^. Microsoft Office software will be utilised to ensure readability recommendations are met ( *i.e.*, grade 6 or less on the Flesch-Kincaid Grade Level) ^41^.

**Focus Group 1**

This focus group will review paper prototypes of the visual feature concepts and the prioritised elements to be developed in further detail for the click-through prototype. Participants will utilise voting technique to determine the value of the individual features. Questions will address adoption, accessibility, and overall strengths/weaknesses of the key features (Appendix B, *Extended data*).

**Focus Group 2**

This focus group will review the initial clickable prototype, with participants being asked to provide feedback on the proposed functional elements and website pages. Questions will focus on functional elements ( *e.g.*, checkboxes, drag and drop, and tailored features) and individual webpage presentations ( *e.g.*, missing items and clarity; Appendix C, *Extended data*). Field notes will be taken in both focus groups. The interview guides for both focus groups will continue to be refined iteratively by research team members.

***Analysis***

The focus groups will be audio recorded, transcribed verbatim and imported into NVivo ^42^. The lead researcher (MK) will complete a descriptive synthesis of the voting results and focus group transcripts. The researchers MK, JMcV and BO’M will then review these findings, and refine the prototype as indicated. 

### Step 2: Heuristic testing

Five reviewers (a panel composed of three research team members [BF, DM & CB], one graphic designer and one software developer) who were not involved in Step 1, will evaluate the refined prototype on a computer and mobile device based on the 10 heuristic principles of Nielsen ^43^. The areas of focus will include search, navigation, forms and data entry, information architecture, writing and content quality, trust and credibility, page layout and visual design.

The reviewers will independently rate each website page using the following criteria: +1 (complies), -1 (does not comply), or 0 (partially complies). Reviewers will provide comments to justify their scoring. For items deemed not relevant, reviewers will be advised to note ‘not applicable’. The reviewers will meet to discuss the ratings and reach a consensus regarding the items to be addressed before usability testing.

### Step 3: Usability testing

***Study participants***

Participants involved in one-to-one interviews will include both individuals with a MSD and MSK physiotherapists.

Individuals with a MSD: Convenience sampling, using the same eligibility criteria and recruitment strategy will be used as described in Step 1. Potential participants will be naïve to the development of the eHealth intervention.

 MSK Physiotherapists: A convenience sampling strategy will also be utilised for MSK physiotherapists. Physiotherapist eligibility criteria include physiotherapists working predominantly in the area of MSK therapy (at least 50% of their time) in either the public or private health setting. To recruit MSK physiotherapists working in the public sector, the researcher MK will contact physiotherapy managers, gatekeepers at these sites, to outline the details of the study; provide a participant information leaflet; and request that an email invitation is sent to all their staff. Email invitations will also be sent out via the ISCP mailing list. The study will also be advertised on social media (Twitter).

Interested participants will be asked to contact the researcher (MK). Those with access to email will be sent a participant information leaflet and consent form. For those that do not have access to a computer or the internet, the participant information leaflet and consent form will be sent via post with a prepaid return envelope. 

Acknowledging that usability testing with 3 to 5 participants can identify 85% of usability issues, we will aim to recruit this number of people with MSDs and MSK physiotherapists ^44^.

***Procedures***

**Questionnaires**

Prior to the interviews, participants will complete the two questionnaires as outlined in Step 1. At the end of the interview, participants will complete the System Usability Scale (SUS) to evaluate user satisfaction ^45^. This validated questionnaire is the most frequently utilised within usability testing ^46^.

**Interviews**

Five usability scenarios for both people with a MSD (Appendix D, *Extended data*) and MSK physiotherapists (Appendix E, *Extended data*) have been developed by the research team. These scenarios will continue to be refined iteratively, as the prototype itself is refined. During the interview, participants will review the relevant five scenarios utilising the think-aloud method ( *i.e.* participants think out aloud while performing a given task) ^47^ and will respond to a series of open-ended questions about the interface, content, features, and format ^48^. The think-aloud method is the most commonly utilised qualitative method of usability testing in eHealth intervention development ^46^, effective in exploring end-users' attitudes towards eHealth interventions ^31, 49^.

The lead researcher (MK) will conduct the one-to-one 60-minute interviews to identify problems with the prototype interface and paths and strategies that participants utilise, including time spent completing tasks. Participants will be interviewed using their preferred devices ( *i.e.*, mobile phone, tablet, laptop and desktop) to review the prototype. At the outset of each interview, informed consent will be re-confirmed orally, and audio-recorded digitally. Field notes will also be taken during each interview.

**Analysis**

Interviews will be audio recorded, transcribed verbatim and exported into NVivo ^42^. The interview transcripts will be analysed using directed content analysis; applying a deductive approach to categorise what was useful and the areas to enhance ^50^. The material will be coded by the lead researcher MK into predefined categories relating to the navigation, content, and design of the prototype. This process will be cross-checked by the research team, with the final list then used by software developers to further refine the eHealth: It’s TIME intervention prototype. A descriptive statistical analysis of the SUS scores will also be completed.

This study will employ a variety of trustworthiness strategies including reflexivity, and data and methodological triangulation. Field notes and memos will serve as tools to facilitate reflexivity ^51^. Regular research team discussions which are open and collegiate ^52^ will also promote reflection on the analysis and results. Collecting data from different sources (i.e., people with MSDs and physiotherapists) using different methodologies (i.e., focus groups, interviews and questionnaires) will allow for data and methodological triangulation ^53^. 

**Strengths and limitations of this study**

The main strength of this study protocol is the user-centred, theory-informed iterative development approach with emphasis on stakeholder engagement to develop and design a multicomponent eHealth-mediated self-management support prototype for people with MSDs. A study limitation is that the prototype will not be implemented and evaluated in clinical practice. Hence, this study will not allow conclusions to be drawn about the actual value of the eHealth-mediated self-management support prototype regarding clinical outcomes and cost-effectiveness. The next step following the conclusion of this study is to test the feasibility of the refined high-fidelity prototype within clinical practice. Another limitation of this study protocol is the use of convenience sampling which may increase the risk of bias towards the prototype. This sampling approach will be undertaken given the limited availability of resources for the conduct of this study. However, to limit this bias, all study participants will only be involved in one aspect of the study, with no prior exposure to the development of the prototype, which will result in a larger overall study sample size. Furthermore, a purposive sampling strategy will be utilised in subsequent development phases. 

## Conclusions

eHealth interventions show potential in supporting self-management of people with MSDs. This study aims to develop and design an eHealth-mediated follow-up self-management support prototype for this cohort. It will be guided by the preferences of end-users, utilising a systematic iterative process. Once developed, the next phase of this project will be to complete a feasibility study to identify preliminary patient-reported outcomes ( *e.g.*, self-efficacy), to evaluate engagement with and the acceptability of the prototype and potential implementation considerations.

## Data Availability

No data are associated with this article. Figshare: Development and design of an eHealth intervention to support self-management in those with musculoskeletal disorders - ‘eHealth: It’s TIME’: a study protocol, ( 10.6084/m9.figshare.23717799) ^53^ This project contains the following extended data: Value Specification Interview Guide Focus Group 1, Interview Guide Focus Group 2 Usability Scenarios for People with a Musculoskeletal Disorder – Usability Testing (Step 3) Usability Scenarios for Musculoskeletal Physiotherapist – Usability Testing (Step 3) Data are available under the terms of the Creative Commons Attribution 4.0 International license (CC-BY 4.0).
