# Supplementary material for: Design and development of an eHealth intervention to support self-management in people with musculoskeletal disorders - ‘eHealth: It’s TIME’: a study protocol
Source: HRB Open Res. 2023 Aug 10;5:73. Originally published 2022 Nov 14. [Version 2] doi: 10.12688/hrbopenres.13611.2 (PMC10477747; doi:10.12688/hrbopenres.13611.2)
Supplement: Peer review history

# Peer review: Reviewer response for version 2

**DOI:** 10.21956/hrbopenres.15078.r35737

**Published:** 2023-09-04

**Authors:** Sara Lundell

**Affiliations:** 1 Department of Sociology, Umea Universitet, Umeå, Västerbotten County, Sweden

I would like to thank the authors for their revisions, which improved the protocol.

Is the study design appropriate for the research question?

Yes

Is the rationale for, and objectives of, the study clearly described?

Yes

Are sufficient details of the methods provided to allow replication by others?

Partly

Are the datasets clearly presented in a useable and accessible format?

Not applicable

Reviewer Expertise:

My area of expertise relevant for this review is conducting co-creation studies to develop eHealth tools for supporting self-management in people with chronic obstructive pulmonary disease (COPD).

I confirm that I have read this submission and believe that I have an appropriate level of expertise to confirm that it is of an acceptable scientific standard.

# Peer review: Reviewer response for version 2

**DOI:** 10.21956/hrbopenres.15078.r35736

**Published:** 2023-08-31

**Authors:** Ingris Pelaez-Ballestas

**Affiliations:** 1 Hospital General de Mexico Dr Eduardo Liceaga, Mexico City, Mexico City, Mexico

I thank the authors for having heeded all the suggestions.

 I believe that with this new version, the manuscript can be indexed.

Is the study design appropriate for the research question?

Yes

Is the rationale for, and objectives of, the study clearly described?

Yes

Are sufficient details of the methods provided to allow replication by others?

Partly

Are the datasets clearly presented in a useable and accessible format?

Not applicable

Reviewer Expertise:

Rheumatology; Qualitative Study; Participatory Research

I confirm that I have read this submission and believe that I have an appropriate level of expertise to confirm that it is of an acceptable scientific standard.

# Peer review: Reviewer response for version 1

**DOI:** 10.21956/hrbopenres.14880.r34398

**Published:** 2023-07-18

**Authors:** Ingris Pelaez-Ballestas

**Affiliations:** 1 Hospital General de Mexico Dr Eduardo Liceaga, Mexico City, Mexico City, Mexico

I appreciate the opportunity to review this interesting and novel protocol “Design and development of an eHealth intervention to support self-management in people with musculoskeletal disorders-eHealth: It´s TIME: a study protocol”. Its implementation will provide support to health professionals and people with MSKD. However, I have some questions and recommendations, especially regarding the methodology.

 Methodology

1. I have doubts about the process of participation in the development. Is it only through the focus groups, or did people with MSKD and physical therapists participate in the study design and construction of the interview guide?
2. It would be important to include a greater diversity of participants regarding gender, ethnicity, place of residence (rural/urban), and socioeconomic conditions.
3. I suggest describing the analysis in detail. Will there be any triangulation strategy?
4. I suggest a reflection on the potential limitations of this protocol or even a reflexivity on the part of the authors.

Is the study design appropriate for the research question?

Yes

Is the rationale for, and objectives of, the study clearly described?

Yes

Are sufficient details of the methods provided to allow replication by others?

Partly

Are the datasets clearly presented in a useable and accessible format?

Not applicable

Reviewer Expertise:

Rheumatology; Qualitative Study; Participatory Research

I confirm that I have read this submission and believe that I have an appropriate level of expertise to confirm that it is of an acceptable scientific standard, however I have significant reservations, as outlined above.

# Peer review: Reviewer response for version 1

**DOI:** 10.21956/hrbopenres.14880.r33542

**Published:** 2023-03-27

**Authors:** Sara Lundell

**Affiliations:** 1 Department of Sociology, Umea Universitet, Umeå, Västerbotten County, Sweden

This is a well-written protocol for a study with the aim to develop and design an eHealth self-management support for people with musculoskeletal disorders. The study will be conducted in three steps; 1) creation of website features and content with feedback from two focus groups, 2) heuristic testing by fiver reviewers, and 3) usability testing with people with musculoskeletal disorders and musculoskeletal physiotherapists. The study design seems highly relevant for the aim of the study.

 Even though I think that the protocol includes most information I would have asked for, I have some suggestions that I think could improve the text even more.

- I think the introduction gives a good and clear motivation on why this study is needed, but please provide a definition/description of self-management.
- Figure 1 gives a great overview of the study, but in contrast to step 1 and 3, it is unclear who the participants, i.e., the five reviewers, in step 2 are. Please clarify that.
- Methods: Please specify if you will have any strategy on who to recruit (beyond eligibility criteria), e.g., distribution between men and women, different ages, computer skills, etc.
- Methods – study design: You state that your study relates to the first three phases of the Roadmap. Please elaborate on how you will do this and how these three steps that you have mentioned earlier in the text.
- Methods – step 1 – study participants: Please specify if the informed consent is written or oral.
- Methods – recruitment: For me it was unclear for me how the interested participants should come in contact with the study, until I read the social media recruitment. Please clarify in the beginning of the recruitment paragraph that interested participants from all these recruitment methods will be asked to contact researcher MK.
- Methods – step 2: The three research team members, have they been part of the development i step 1? Please specify.
- Methods – step 3 – study participants: Please, revise the sentence about purposive sampling of individuals with a MSD.
- Methods – step 3 – study participants: The last sentence of the last paragraph should be moved to the paragraph about MSK Physiotherapists.
- Methods – step 3 – procedures – questionnaire: Please look at the last sentence, I think it needs to be rephrased.
- Methods – step 3 – procedures – interviews: I would prefer to get the information about the think-aloud method earlier in the text. Now several questions were raised before I got to that part. Please restructure this part.

Is the study design appropriate for the research question?

Yes

Is the rationale for, and objectives of, the study clearly described?

Yes

Are sufficient details of the methods provided to allow replication by others?

Partly

Are the datasets clearly presented in a useable and accessible format?

Not applicable

Reviewer Expertise:

My area of expertise relevant for this review is conducting co-creation studies to develop eHealth tools for supporting self-management in people with chronic obstructive pulmonary disease (COPD).

I confirm that I have read this submission and believe that I have an appropriate level of expertise to confirm that it is of an acceptable scientific standard, however I have significant reservations, as outlined above.
